# Acute ischemic stroke in tuberculous meningitis

**DOI:** 10.3389/fpubh.2024.1362465

**Published:** 2024-03-21

**Authors:** Yi-Jia Guo, Xin-Ling Gan, Ru-Yun Zhang, Yong Liu, Er-Li Wang, Shui-Hua Lu, Hui Jiang, Hong-Fei Duan, Zheng-Zhou Yuan, Wei-Min Li

**Affiliations:** ^1^Beijing Chest Hospital, Capital Medical University, Beijing, China; ^2^Beijing Municipal Key Laboratory of Clinical Epidemiology, School of Public Health, Capital Medical University, Beijing, China; ^3^Department of Neurology, The First Affiliated Hospital of Chengdu Medical College, Chengdu, China; ^4^Key Laboratory of Rehabilitation Medicine in Sichuan Province, West China Hospital, Sichuan University, Chengdu, China; ^5^Department of Emergency, Beijing Chest Hospital, Capital Medical University, Beijing, China; ^6^Department of Radiology, The First People's Hospital of Longquanyi District, Chengdu, China; ^7^National Clinical Research Center for Infectious Diseases, Guangdong Provincial Clinical Research Center for Tuberculosis, Shenzhen Third People's Hospital, Southern University of Science and Technology, Shenzhen, China; ^8^Department of Tuberculosis, Beijing Chest Hospital, Capital Medical University, Beijing Tuberculosis and Thoracic Tumor Research Institute, Beijing, China; ^9^Department of Neurology, The Affiliated Hospital of Southwest Medical University, Luzhou, China; ^10^National Tuberculosis Clinical Lab of China, Beijing Key Laboratory in Drug Resistance Tuberculosis Research, Beijing Tuberculosis and Thoracic Tumor Research Institute, Beijing, China

**Keywords:** tuberculous meningitis, acute ischemic stroke, cohort study, risk factor, mediation analysis

## Abstract

**Background:**

The underlying mechanism for stroke in patients with tuberculous meningitis (TBM) remains unclear. This study aimed to investigate the predictors of acute ischemic stroke (AIS) in TBM and whether AIS mediates the relationship between inflammation markers and functional disability.

**Methods:**

TBM patients admitted to five hospitals between January 2011 and December 2021 were consecutively observed. Generalized linear mixed model and subgroup analyses were performed to investigate predictors of AIS in patients with and without vascular risk factors (VAFs). Mediation analyses were performed to explore the potential causal chain in which AIS may mediate the relationship between neuroimaging markers of inflammation and 90-day functional outcomes.

**Results:**

A total of 1,353 patients with TBM were included. The percentage rate of AIS within 30 days after admission was 20.4 (95% CI, 18.2–22.6). A multivariate analysis suggested that age ≥35 years (OR = 1.49; 95% CI, 1.06–2.09; *P* = 0.019), hypertension (OR = 3.56; 95% CI, 2.42–5.24; *P* < 0.001), diabetes (OR = 1.78; 95% CI, 1.11–2.86; *P* = 0.016), smoking (OR = 2.88; 95% CI, 1.68–4.95; *P* < 0.001), definite TBM (OR = 0.19; 95% CI, 0.06–0.42; *P* < 0.001), disease severity (OR = 2.11; 95% CI, 1.50–2.90; *P* = 0.056), meningeal enhancement (OR = 1.66; 95% CI, 1.19–2.31; *P* = 0.002), and hydrocephalus (OR = 2.98; 95% CI, 1.98–4.49; *P* < 0.001) were associated with AIS. Subgroup analyses indicated that disease severity (P for interaction = 0.003), tuberculoma (P for interaction = 0.008), and meningeal enhancement (P for interaction < 0.001) were significantly different in patients with and without VAFs. Mediation analyses revealed that the proportion of the association between neuroimaging markers of inflammation and functional disability mediated by AIS was 16.98% (95% CI, 7.82–35.12) for meningeal enhancement and 3.39% (95% CI, 1.22–6.91) for hydrocephalus.

**Conclusion:**

Neuroimaging markers of inflammation were predictors of AIS in TBM patients. AIS mediates < 20% of the association between inflammation and the functional outcome at 90 days. More attention should be paid to clinical therapies targeting inflammation and hydrocephalus to directly improve functional outcomes.

## Introduction

Tuberculous meningitis (TBM) is the most severe form of tuberculosis (TB) infection, which is responsible for approximately 40% of TB-associated deaths in developing countries (1). Acute ischemic stroke (AIS) is one of the common complications following TBM (2, 3). It has been reported that the incidence of AIS in TBM patients is approximately 30% (4), the possible mechanism for AIS likely due to chronic vasculitis, intimal proliferation, and the hypercoagulable state (2, 5). The development of AIS in TBM patients can cause irreversible neurological deficits and can lead to functional disability (4). It is of great importance to identify TBM patients at high risk of AIS to prevent further disease progression. However, the risk factors for AIS are not fully understood in TBM patients.

There have been several observational studies that have attempted to determine the predictors of stroke in TBM (6–11), but these were single-center studies with small sample sizes, and the conclusion seemed to vary across studies conducted in different countries (4). Moreover, most stroke mimics and imaging presentations appeared on admission, and the timing of the onset of stroke in TBM was not clear in previous reports (4, 12). Predictive factors, such as clinical presentation, laboratory parameters, and neuroimaging, could be assessed after the onset of stroke. Therefore, these results need to be interpreted with caution because the demonstration of a temporal relation cannot be achieved (12). Moreover, for a stroke of rare etiology, such as TBM, conventional risk factors may not fully account for the pathogenesis of stroke; hence, more research is required to fill the research gaps in this field.

In the present study, we aimed to determine the predictors associated with AIS within 30 days after admission in a multicenter TBM cohort and analyze the difference in patients with and without vascular risk factors (VAFs). Moreover, we further explored the extent to which AIS mediated the relationships between inflammation markers and functional disability at 90 days in TBM patients and assessed the mediation effect.

## Methods

### Study population

The present study was a retrospective cohort analysis carried out from January 2011 to December 2021 in five cooperative hospitals. According to the standard diagnostic criteria (13), patients with a confirmed or clinical diagnosis of TBM attending local hospitals were consecutively observed. Definite TBM was confirmed by the presence of acid-fast bacilli by cerebrospinal fluid (CSF) microscopy or *Mycobacterium tuberculosis* cultured from the CSF. Probable and possible TBM was diagnosed based on the diagnostic criteria score (13). The exclusion criteria were as follows: patients with fungal or other bacterial infections; patients with autoimmune or other immune diseases; patients with a history of previous stroke; and patients with missing clinical or imaging data. This study was approved by the ethics committee at Beijing Chest Hospital. Informed consent was exempted by the ethics committee because of the retrospective nature of this study (approval number: YJS-2022-07). The study protocol was reported in accordance with the “Strengthening the Reporting of Observational Studies in Epidemiology” (STROBE) guidelines (14).

### Data collection

Demographic features, comorbidities (hypertension, diabetes mellitus, hyperlipidemia, and atrial fibrillation), clinical manifestations, laboratory parameters, imaging examinations (such as brain CT/MRI, diffusion-weighted MRI, chest CT, or X-ray), and clinical treatments were collected. The presence of tuberculoma, basal meningeal enhancement, hydrocephalus, and AIS was identified by two well-trained neurologists, and inconsistencies were resolved by a senior neurologist. Data on peripheral markers of inflammation, such as white blood cells, neutrophils, monocytes, lymphocytes, platelets, and C-reactive protein, were collected within 24 h after admission, before the initiation of the treatment. The first CSF microscopy result was obtained for pressure, cells, glucose, chloride, and protein. The severity of meningitis was staged based on the British Medical Research Council (BMRC) guidelines at admission (15). The BMRC grade included two main dimensions: impaired consciousness status and neurological deficits; a clinical severity between grades I and III was given for diagnosis.

### Follow-up and AIS event

Patients were monitored for clinical symptoms of neurological deficit for 30 days after admission. The diagnosis of AIS was defined according to the AHA/ASA guidelines and confirmed by medical records (16). Definite stroke diagnosis was made based on patients who had recently suffered from focal neurological deficit and who had undergone brain MRI that identified an ischemic infarct, which is consistent with the clinical symptoms. After discharge, the patients were followed up on the 90th day at local clinical centers. The neurological deficit was assessed by a modified Rankin Scale (mRS). The functional disability was defined as an mRS score of 3–6.

### Statistical analyses

Descriptive characteristics were reported as percentages for categorical variables or as mean with standard deviation for continuous variables. χ^2^ test or Fisher's exact test, Student's *t*-test, or the Mann–Whitney *U* test were performed for statistical analysis when appropriate. A two-level generalized linear mixed model (GLMM) was fitted using a logit link to identify the associated factors of AIS (with patients being at the first level and hospitals at the second level) to account for the expected correlation in outcomes within different centers. Subgroup analyses were performed by using a test for interaction in patients with and without VAFs (hypertension, diabetes mellitus, hyperlipidemia, and atrial fibrillation). The net reclassification index (NRI) and integrated discrimination index (IDI) were calculated to quantify the improvement in the correct reclassification and sensitivity with the addition of neuroimaging markers of inflammation to stroke prediction tools (17, 18). Significant improvement was observed in the prediction model when NRI was >0 or IDI was >0. To reduce the effect of confounding factors, we performed propensity score matching (PSM) and inverse probability of treatment weighting (IPTW) as confirmatory analyses to determine the association between neuroimaging markers of inflammation and AIS. Further causal mediation analysis using a counterfactual framework was performed to explore whether AIS mediates the relationship between inflammation markers and functional disability. All analyses were conducted using R version 4.2.0.

## Results

### Baseline characteristics

**Figure 1:**
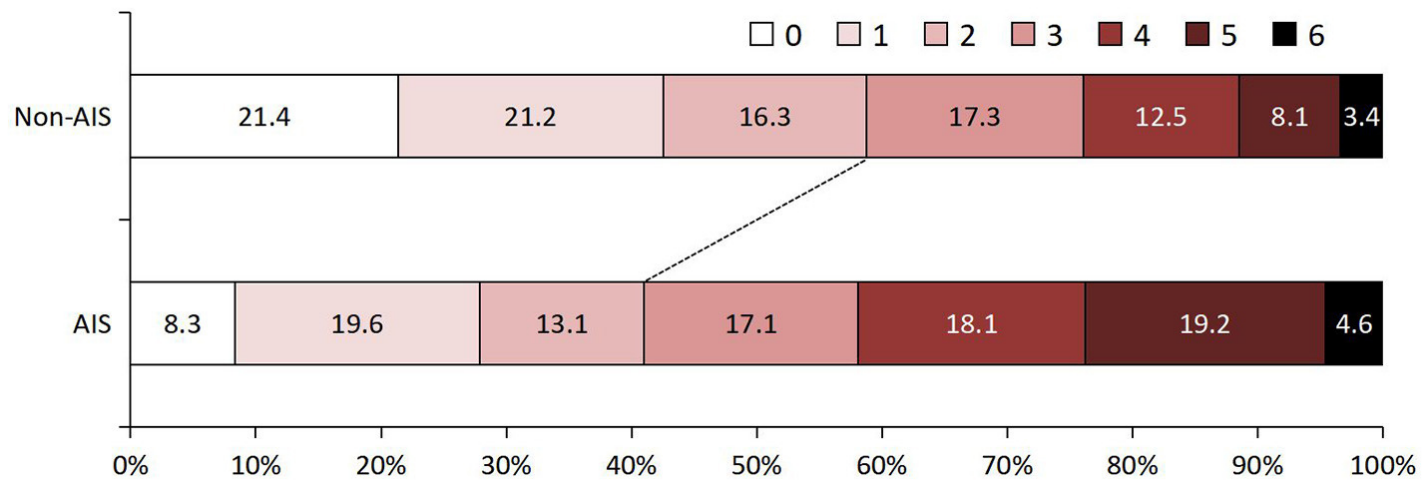
Ninety-day functional outcome assessed by modified Rankin Scale in TB patients with and without AIS. AIS, acute ischemic stroke; TBM, tuberculous meningitis.

The study flow chart is shown in Supplementary Figure S1. At baseline, there were 1,686 patients with a diagnosis of TBM. After excluding patients with fungal or other bacterial infections (*n* = 106), patients with autoimmune or other immune diseases (*n* = 43), patients with a history of previous stroke (*n* = 86), and patients with missing data (*n* = 98), a total of 1,353 patients were analyzed in the study. The mean age was 38.8 (±18.3) years, and 53.0% of the patients were men. Of these patients, 275 (20.4%) had a diagnosis of AIS during the 30 days after admission. Information on baseline characteristics and clinical treatment of participants with and without AIS is depicted in Table 1. Age, VAFs (hypertension, diabetes, and smoking), diagnosis classification, BMRC grade, CT/MRI findings (tuberculoma, meningeal enhancement, and hydrocephalus), CSF leukocyte count, and peripheral inflammation parameters (white blood cells, monocytes, lymphocytes, and platelets) were significantly different in both groups (*P* < 0.05). TBM patients who developed AIS had a higher proportion of 90-day follow-up functional disability (Figure 1).

**Table 1:** Baseline information of study participants with 30-day AIS.

**Variables**	**Overall (*n =* 1,353)**	**Without AIS (*n =* 1,078)**	**With AIS (*n =* 275)**	***P***
**Demographics**				
Age, y	38.8 ± 18.3	37.3 ± 17.6	44.9 ± 19.7	< 0.001
Men	718 (53.0)	582 (54.0)	136 (49.5)	0.179
Body mass index, kg/m^2^	21.6 ± 3.0	21.5 ± 2.4	21.6 ± 3.1	0.613
**Vascular risk factors**				
Hypertension	185 (13.6)	112 (10.4)	73 (26.5)	< 0.001
Diabetes	158 (11.6)	113 (10.5)	45 (16.4)	0.007
Hyperlipidemia	151 (11.1)	118 (10.9)	33 (12.0)	0.620
Atrial fibrillation	45 (3.3)	37 (3.4)	8 (2.9)	0.666
Smoking	85 (6.2)	58 (5.4)	27 (9.8)	0.008
**Clinical characteristics**				
**Diagnosis classification**				< 0.001
Probable TBM	334 (24.6)	262 (24.3)	72 (26.2)	
Possible TBM	840 (62.0)	646 (59.9)	194 (70.5)	
Definite TBM	179 (13.2)	170 (15.8)	9 (3.3)	
**BMRC grade**				< 0.001
Stage I	683 (50.4)	573 (53.2)	110 (40.0)	
Stage II	495 (36.5)	369 (34.2)	126 (45.8)	
Stage III	175 (12.9)	136 (12.6)	39 (14.2)	
**CT/MRI findings**				
Pulmonary tuberculosis	964 (71.2)	767 (71.2)	197 (71.6)	0.874
Tuberculoma	588 (43.4)	451 (41.8)	137 (49.8)	0.017
Meningeal enhancement	363 (26.8)	269 (25.0)	94 (34.2)	0.002
Hydrocephalus	152 (11.2)	96 (8.9)	56 (20.4)	< 0.001
**Cerebrospinal fluid**				
Pressure, mm H_2_0	239.9 ± 77.2	239.4 ± 75.0	241.9 ± 85.3	0.867
Leukocyte count, 10^6^/L	203.8 ± 257.3	205.6 ± 247.5	196.3 ± 292.6	0.036
Glucose, mmol/L	2.2 ± 1.2	2.2 ± 1.3	2.0 ± 0.9	0.147
Chloride, mmol/L	112.9 ± 7.6	112.8 ± 7.7	113.3 ± 7.1	0.442
Protein, mg/dl	155.6 ± 92.4	158.0 ± 96.8	145.9 ± 72.4	0.221
**Peripheral blood parameters**				
White blood cells, 10^9^/L	7.4 ± 2.3	7.5 ± 3.1	6.9 ± 2.7	0.003
Neutrophils, 10^9^/L	5.5 ± 2.1	5.5 ± 2.8	5.3 ± 2.6	0.088
Monocytes, 10^9^/L	0.5 ± 0.2	0.5 ± 0.5	0.4 ± 0.3	< 0.001
Lymphocytes, 10^9^/L	1.2 ± 0.5	1.3 ± 0.8	1.1 ± 0.6	< 0.001
Platelets, 10^9^/L	243.2 ± 65.0	246.0 ± 81.0	232.0 ± 98.0	< 0.001
C-reactive protein, mg/L	18.4 ± 19.3	18.0 ± 26.7	18.5 ± 29.5	0.107
**Treatment**				
Isoniazid	1,306 (96.5)	1,045 (96.9)	261 (94.9)	0.101
Rifampicin	1,160 (85.7)	918 (85.4)	242 (88.0)	0.132
Pyrazinamide	1,321 (97.6)	1,051 (97.5)	270 (98.2)	0.504
Ethambutol	1,092 (80.7)	878 (81.4)	214 (77.8)	0.173
Fluoroquinolones	445 (32.8)	350 (32.5)	95 (34.5)	0.513
Other anti-tuberculosis drugs	189 (13.9)	143 (13.3)	46 (16.7)	0.139
Intravenous dexamethasone	792 (58.5)	635 (58.9)	157 (57.1)	0.949
Intrathecal injection	868 (64.1)	688 (63.8)	180 (65.5)	0.178
Dehydration treatment	1,264 (93.4)	1,018 (94.4)	246 (89.5)	0.145
Shunt surgery	31 (2.2)	24 (2.2)	7 (2.5)	0.703

AIS, acute ischemic stroke; TBM, tuberculous meningitis; BMRC, British Medical Research Council.

### GLMM analysis of associated factors with AIS

The percentage rate of AIS within 30 days after admission was 20.4 (95% CI, 18.2–22.6). A univariate analysis in GLMM showed that (Table 2) showed that age ≥35 years (OR = 1.68; 95% CI, 1.27–2.22; *P* < 0.001), hypertension (OR = 3.23; 95% CI, 2.32–4.51; *P* < 0.001), diabetes (OR = 1.73; 95% CI, 1.19–2.51; *P* = 0.004), smoking (OR = 1.97; 95% CI, 1.22–3.18; *P* = 0.005), definite TBM (OR = 0.18; 95% CI, 0.08–0.36; *P* < 0.001), disease severity (OR = 1.69; 95% CI, 1.29–2.45; *P* < 0.001 for BMRC Stage II; OR = 1.51; 95% CI, 0.98–2.32; *P* = 0.059 for Stage III), tuberculoma (OR = 1.46; 95% CI, 1.06–1.90; *P* = 0.016), meningeal enhancement (OR = 1.63; 95% CI, 1.22–2.17; *P* = 0.001), hydrocephalus (OR = 2.71; 95% CI, 1.89–3.88; *P* < 0.001), CSF glucose level (OR = 0.89; 95% CI, 0.79–1.01; *P* = 0.059), peripheral blood white blood cells (OR = 0.92; 95% CI, 0.88–0.96; *P* = 0.008), monocytes (OR = 0.43; 95% CI, 0.26–0.75; *P* = 0.003), lymphocytes (OR = 0.68; 95% CI, 0.56–0.85; *P* < 0.001), and platelets (OR = 0.99; 95% CI, 0.99–1.00; *P* = 0.018).

**Table 2:** Generalized linear mixed model analysis of associated factors with 30-day AIS.

**Variables**	**Univariate analysis**			**Multivariate analysis**		
	**OR**	**95% CI**	***P***	**OR**	**95% CI**	***P***
**Demographics**						
Age ≥ 35, y	1.68	1.27–2.22	< 0.001	1.49	1.06–2.09	0.019
Men	0.78	0.60–1.03	0.082			
Body mass index ≥ 24, kg/m^2^	0.99	0.95–1.03	0.572			
**Vascular risk factors**						
Hypertension	3.23	2.32–4.51	< 0.001	3.56	2.42–5.24	< 0.001
Diabetes	1.73	1.19–2.51	0.004	1.78	1.11–2.86	0.016
Hyperlipidemia	1.15	0.76–1.73	0.520			
Atrial fibrillation	0.82	0.32–1.78	0.612			
Smoking	1.97	1.22–3.18	0.005	2.88	1.68–4.95	< 0.001
**Clinical characteristics**						
**Diagnosis classification**						
Probable TBM			Reference			Reference
Possible TBM	1.02	0.78–1.39	0.496	1.18	0.88–1.66	0.162
Definite TBM	0.18	0.08–0.36	< 0.001	0.19	0.06–0.42	< 0.001
**BMRC**						
Stage I			Reference			Reference
Stage II	1.69	1.29–2.45	< 0.001	2.11	1.50–2.90	0.056
Stage III	1.51	0.98–2.32	0.059	1.25	0.93–1.76	0.168
**CT/MRI features**						
Pulmonary tuberculosis	0.98	0.73–1.32	0.921			
Tuberculoma	1.46	1.11–1.90	0.006	1.28	0.95–1.66	0.128
Meningeal enhancement	1.63	1.22–2.17	0.001	1.66	1.19–2.31	0.002
Hydrocephalus	2.71	1.89–3.88	< 0.001	2.98	1.98–4.49	< 0.001
**Cerebrospinal fluid parameters**						
Pressure, mm H_2_0	1.00	0.99–1.00	0.860			
Leukocyte count, 10^6^/L	1.00	0.99–1.00	0.508			
Glucose, mmol/L	0.89	0.79–1.01	0.059	0.93	0.84–1.09	0.113
Chloride, mmol/L	1.01	0.99–1.03	0.360			
Protein, mg/dl	0.99	0.99–1.00	0.110			
**Peripheral blood parameters**						
White blood cells, 10^9^/L	0.92	0.88–0.96	0.004	0.98	0.90–1.03	0.110
Neutrophils, 10^9^/L	0.95	0.91–1.03	0.159			
Monocytes, 10^9^/L	0.43	0.26–0.75	0.003	0.70	0.35–1.02	0.089
Lymphocytes, 10^9^/L	0.68	0.56–0.85	< 0.001	0.95	0.75–1.19	0.639
Platelets, 10^9^/L	0.99	0.99–1.00	0.018	0.99	0.99–1.00	0.250
C-reactive protein, mg/L	0.99	0.99–1.00	0.906			
**Treatment**						
Isoniazid	0.59	0.31–1.12	0.105			
Rifampicin	1.22	0.70–2.02	0.135			
Pyrazinamide	1.39	0.53–3.64	0.506			
Ethambutol	0.79	0.58–1.10	0.174			
Fluoroquinolones	1.10	0.83–1.45	0.513			
Other anti-tuberculosis drugs	1.25	0.82–1.87	0.144			
Intravenous dexamethasone	0.96	0.86–1.14	0.949			
Intrathecal injection	1.08	0.73–1.54	0.109			
Dehydration treatment	0.69	0.42–1.14	0.147			
Shunt surgery	1.18	0.50–2.77	0.704			

AIS, acute ischemic stroke; TBM, tuberculous meningitis; BMRC, British Medical Research Council.

After adjusting for covariates with a *p*-value of < 0.05, a multivariate analysis indicated that age ≥35 (OR = 1.49; 95% CI, 1.06–2.09; *P* = 0.019), hypertension (OR = 3.56; 95% CI, 2.42–5.24; *P* < 0.001), diabetes (OR = 1.78; 95% CI, 1.11–2.86; *P* = 0.016), smoking (OR = 2.88; 95% CI, 1.68–4.95; *P* < 0.001), definite TBM (OR = 0.19; 95% CI, 0.06–0.42; *P* < 0.001), disease severity (OR = 2.11; 95% CI, 1.50–2.90; *P* = 0.056 for BMRC Stage II), meningeal enhancement (OR = 1.66; 95% CI, 1.19–2.31; *P* = 0.002), and hydrocephalus (OR = 2.98; 95% CI, 1.98–4.49; *P* < 0.001) were determined to be independent predictive factors of AIS (Table 2).

**Figure 2:**
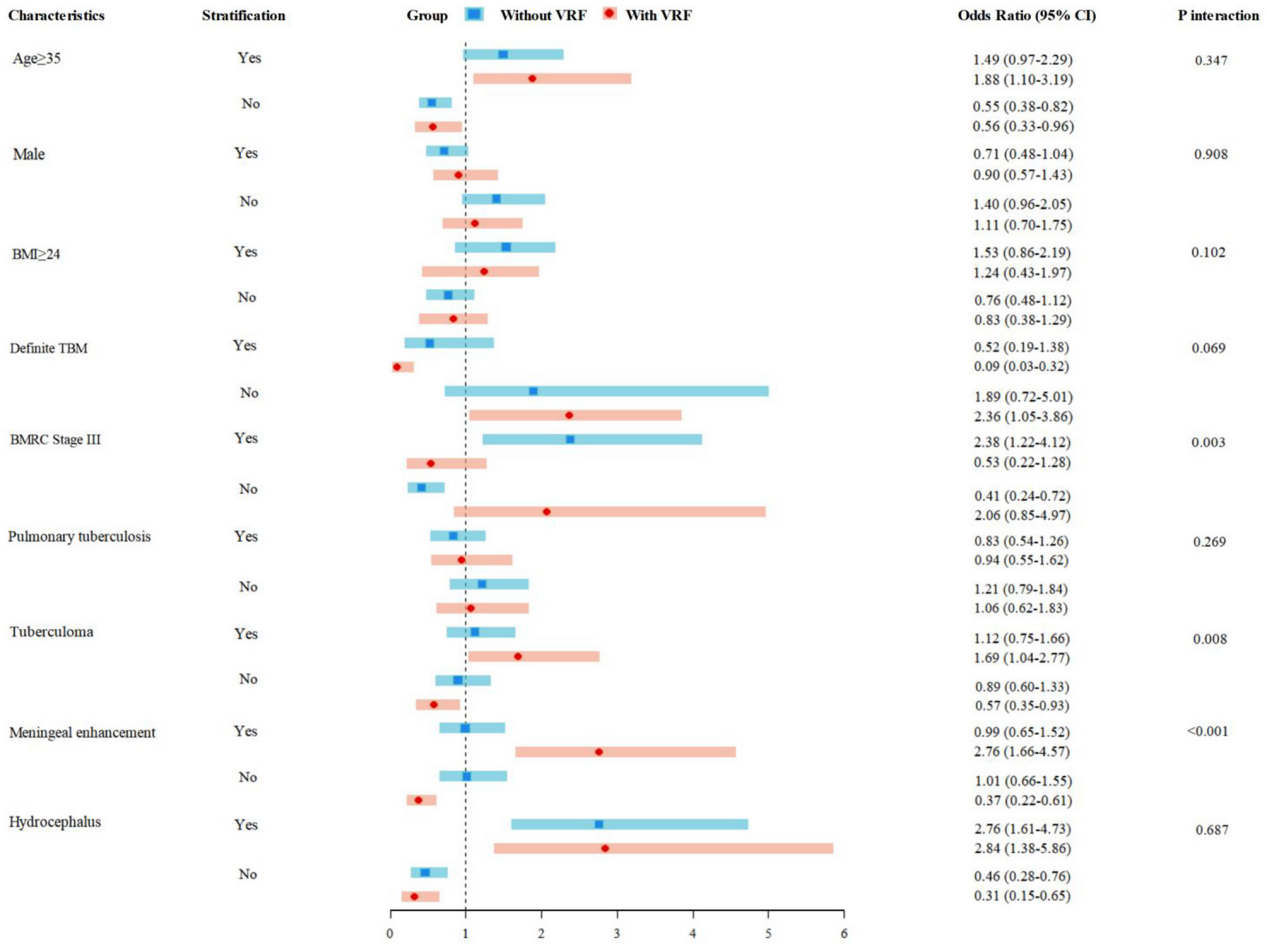
AIS risk in TBM patients with and without conventional VAFs. AIS, acute ischemic stroke; TBM, tuberculous meningitis; VAFs, vascular risk factors.

Subgroup difference analyses further explored associated factors related to AIS in patients with and without VAFs, and the results suggest that significant differences were observed in advanced severity (BMRC Stage III, P for interactio*n* = 0.003), tuberculoma (P for interactio*n* = 0.008), and meningeal enhancement (P for interaction < 0.001). Age ≥ 35 years and hydrocephalus were significant predictors associated with AIS both in VAF and without-VAF subgroups (Figure 2).

### PSM and IPTW analysis demonstrated the association between neuroimaging markers of inflammation and AIS

After adjusting by PSM and IPTW to reduce the effect of confounding factors other than neuroimaging markers, including tuberculoma, meningeal enhancement, and hydrocephalus, the two groups were found to be well-balanced (Supplementary Tables S1–S3). The association between neuroimaging markers and AIS event is summarized in Table 3. After PSM and IPTW analyses, meningeal enhancement (OR = 1.45, 95% CI, 1.00–2.04, *P* = 0.051 for PSM; OR = 1.76, 95% CI, 1.46–2.05, *P* < 0.001 for IPTW) and hydrocephalus (OR = 4.06, 95% CI, 2.19–7.46, *P* < 0.001 for PSM; OR = 2.78, 95% CI, 2.36–3.38, *P* < 0.001 for IPTW) were significantly associated with AIS in patients with TBM.

**Table 3:** Confirmatory analysis of the association between neuroimaging markers of inflammation and 30-day AIS.

**Neuroimaging signs**	**Group**	**Crude**			**GLMM**			**PSM**			**IPTW**		
		**Event/N**	**OR (95% CI)**	***P***	**Event/N**	**OR (95% CI)**	***P***	**Event/ N**	**OR (95% CI)**	***P***	**Event/N**	**OR (95% CI)**	***P***
Tuberculoma	Yes	137/588	1.38 (1.06–1.80)	0.017	137/588	1.28 (0.95–1.66)	0.128^a^	100/404	1.20 (0.88–1.69)	0.358^b^	301/1,279	1.38 (1.10–1.59)	0.004^c^
	No	138/765						89/404			256/1,344		
Meningeal enhancement	Yes	94/363	1.56 (1.17–2.08)	0.002	94/363	1.66 (1.19–2.31)	0.002^a^	87/326	1.45 (1.00–2.04)	0.051^b^	368/1,340	1.76 (1.46–2.05)	< 0.001^c^
	No	181/990			181/990			66/326			242/1,357		
Hydrocephalus	Yes	56/152	2.62 (1.82–3.75)	< 0.001	56/152	2.98 (1.98–4.49)	< 0.001^a^	53/125	4.06 (2.19–7.46)	< 0.001^b^	473/1,248	2.78 (2.36–3.38)	< 0.001^c^
	No	219/1,201			219/1,201			19/125			244/1,353		

GLMM, generalized linear mixed model; PSM, propensity score matching; IPTW, inverse probability of treatment weighting. ^a^*P*-value by a generalized linear mixed model fitted with logistic regression. ^b^*P*-value by conditional logistic regression. ^c^*P*-value by weighted logistic regression.

### Discrimination and calibration analysis of neuroimaging markers of inflammation added to predictive models of AIS

We used stroke risk stratification models to classify patients as low risk, medium risk, and high risk on admission. The prevalence of high risk identified by stroke stratification models ranged from 10.13% to 13.67% (Supplementary Table S4). Compared to other neuroimaging markers, hydrocephalus combined with high-risk stratification showed a better improvement in concordance with (C)-statistic in the prediction of an AIS event, as confirmed by the NRI and IDI. The addition of the hydrocephalus to stroke risk stratification models did contribute to an increase in C-statistic, which ranged from 0.7192 to 0.7764 (*P* < 0.001) for model 1, 0.7573 to 0.8060 (*P* < 0.001) for model 2, and 0.7460 to 0.7954 (*P* < 0.001) for model 3. Reclassification combined with the hydrocephalus also displayed the following: model 1 (NRI, 11.46%, *P* < 0.001; IDI: 2.07%, *P* < 0.001), model 2 (NRI, 11.67%, *P* < 0.001; IDI: 2.28%, *P* < 0.001), and model 3 (NRI, 11.55%, *P* < 0.001; IDI: 2.16%, *P* < 0.001). The results of C-statistic, NRI, and IDI are shown in Table 4.

**Table 4:** Performance of predictive models with neuroimaging markers of inflammation for 30-day AIS.

	**C-statistic**		**Category NRI**		**IDI**	
	**Estimate (95% CI)**	***P***	**Estimate (95% CI)**	***P***	**Estimate (95% CI)**	***P***
**Model 1**						
Basic model	0.7192 (0.6949–0.7434)	Reference		Reference		Reference
Basic model +Tuberculoma	0.7475 (0.7122–0.7827)	0.035	4.30 (-2.26–10.85)	0.198	0.58 (0.17–0.98)	0.005
Basic model +Meningeal enhancement	0.7559 (0.7255–0.7941)	0.022	7.17 (1.17–13.17)	0.019	0.81 (0.30–1.31)	0.002
Basic model +Hydrocephalus	0.7764 (0.7449–0.8077)	< 0.001	11.46 (6.40–16.51)	< 0.001	2.07 (1.14–3.01)	< 0.001
**Model 2**						
Basic model	0.7573 (0.7317–0.7828)		Reference		Reference	
Basic model +Tuberculoma	0.7983 (0.7629–0.8337)	0.002	7.98 (1.38–14.58)	0.018	0.84 (0.34–1.35)	0.001
Basic model +Meningeal enhancement	0.7942 (0.7595–0.8288)	0.004	9.23 (3.06–1.54)	0.003	1.01 (0.43–1.57)	< 0.001
Basic model +Hydrocephalus	0.8060 (0.7746–0.8373)	< 0.001	11.67 (6.61–16.72)	< 0.001	2.28 (1.29–3.27)	< 0.001
**Model 3**						
Basic model	0.7460 (0.7221–0.7699)		Reference		Reference	
Basic model +Tuberculoma	0.7754 (0.7396–0.8112)	0.034	7.96 (1.35–14.56)	0.017	0.87 (0.37–1.36)	< 0.001
Basic model +Meningeal enhancement	0.7831 (0.7488–0.8174)	0.005	9.22 (3.05–1.52)	0.004	0.93 (0.38–1.48)	< 0.001
Basic model +Hydrocephalus	0.7954 (0.7646–0.8262)	< 0.001	11.55 (6.49–16.60)	< 0.001	2.16 (1.20–3.13)	< 0.001

NRI, Net Reclassification Improvement; IDI, Integrated Discrimination Improvement; Model 1, Atherosclerotic Cardiovascular Disease Risk Model recommended by the European Society of Cardiology; Model 2, Stroke risk score card recommended by the Stroke Prevention Committee of the Chinese National Health Commission; Model 3, Framingham Stroke Risk Profile.

### The association between neuroimaging markers of inflammation and 90-day functional disability mediated by AIS

**Figure 3:**
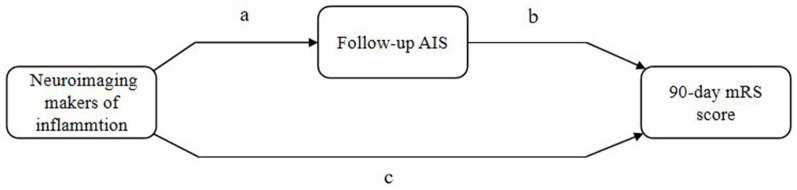
Illustration of mediation effect. AIS, acuter ischemic stroke; mRS, modified Rankin Scale. Total effect = natural direct effect (c) + natural indirect effect (ab).

Mediation analyses within the counterfactual framework were performed to illustrate the mediation effect (Figure 3). Table 5 shows the total, direct, and indirect associations between meningeal enhancement and hydrocephalus with functional disability. Mediation analyses in the adjusted model revealed that the proportion of the association between the neuroimaging markers of inflammation and functional disability mediated by AIS was 16.98% (95% CI, 7.82%−35.12%) for meningeal enhancement and 3.39% (95% CI, 1.22%−6.91%) for hydrocephalus.

**Table 5:** The effect of inflammation-associated neuroimaging markers on 90-day disability mediated by AIS.

	**Unadjusted analysis**		**Adjusted analysis** ^**a**^	
	**Estimate (95% CI)**	***P***	**Estimate (95% CI)**	***P***
**Meningeal enhancement mediated by AIS on 90-day disability, %**				
Total effect	9.75 (3.93–15.20)	< 0.001	9.12 (4.40–14.36)	< 0.001
Natural direct effect	8.34 (2.62–14.39)	< 0.001	7.65 (2.95–12.45)	< 0.001
Natural indirect effect	1.41 (0.52–3.89)	< 0.001	1.46 (0.73–2.54)	< 0.001
Percentage-mediated	14.01 (5.19–36.02)	< 0.001	16.72 (7.42–34.06)	< 0.001
**Hydrocephalus mediated by AIS on 90-day disability, %**				
Total effect	49.63 (44.68–54.32)	< 0.001	39.70 (31.66–46.02)	< 0.001
Natural direct effect	47.61 (42.05–52.42)	< 0.001	38.02 (29.34–45.63)	< 0.001
Natural indirect effect	2.02 (0.84–4.04)	< 0.001	1.68 (0.64–3.10)	< 0.001
Percentage-mediated	2.54 (0.92–6.08)	< 0.001	3.45 (1.26–7.03)	< 0.001

^a^Confounders with a *p-*value of < 0.05 examined in the causal mediation analysis included demographic and vascular risk factors (age, diabetes, and atrial fibrillation), TBM diagnosis classification, BMRC grade, pulmonary tuberculosis, laboratory data (cerebrospinal fluid pressure, peripheral white blood cells, neutrophils, lymphocytes, platelets, and C-reactive protein), and clinical treatment. TBM, tuberculous meningitis; BMRC, British Medical Research Council.

## Discussion

In the present study, we found that age, disease severity, meningeal enhancement, and hydrocephalus were independent predictors of stroke in TBM patients. Subgroup analysis results suggested that VAF modifies the effect of inflammation on AIS risk. Adding these inflammation-associated neuroimaging markers to stratification models improved the prediction of AIS. Our findings also revealed that the proportion of the association between neuroimaging markers of inflammation and functional disability mediated by AIS was 16.98% for meningeal enhancement and 3.39% for hydrocephalus.

Affected by TB burden, the proportions of stroke in patients with TBM varied across different countries and regions (4, 19). A recent systematic review demonstrated that the frequency of stroke in TBM was estimated to be 30% across the world (4). In five studies involving 722 Chinese patients with stroke in TBM (4), the estimated frequency of stroke is 27%, which is higher than the observed frequency of 20% in our data. The onset of stroke in TBM patients may present as silent or asymptomatic (11). The present retrospective cohort analysis only included the new-onset AIS patients, which may justify the low percentage of stroke cases in our data.

Previous studies showed that factors such as older age (6, 11), disease severity (10), peripheral markers of inflammation (10), and CSF cytokines (7) are associated with stroke in patients with TBM, suggesting that the host immune status may be related to the development of stroke. The diversity of neuroimaging markers of inflammation is probably the representative of the host immune response in TBM (1). Tuberculoma is the most common neuroimaging manifestation, which presents in approximately 50% of TBM patients. It could restrict the growth of *Mycobacterium tuberculosis* and reduce the inflammatory response when brain parenchymal infection occurs. The association between tuberculoma and stroke remains controversial. In a cohort of young adults with TBM, tuberculoma was determined to be negatively related to stroke (11). Another two studies showed that the presence of tuberculoma was similar between TBM patients with and without stroke (9, 20). In the present study, we found that tuberculoma was common in the AIS group and that the association between tuberculoma and AIS was more notable in TBM patients with VAFs. We speculated the possible reason that VRF affects the inflammatory process in TBM patients with tuberculoma and increases the risk of stroke (21). In addition, our result showed that meningeal enhancement was an independent predictor of stroke in patients with TBM, which is consistent with the findings of previous studies (11, 12). Meningeal enhancement may portend individuals with a more intensive inflammation response, which may aggravate the process of vasculitis, intimal proliferation, and hypercoagulation (2, 5). In a further subgroup analysis, we observed that the odds ratio of meningeal enhancement was more significant in TBM patients with VAFs than in those without VAFs. This finding indicates the presence of an inflammation–VAF interaction. A recent study has demonstrated the combined effect of inflammation and VAFs on the mechanism of ischemic disease (21). This result may explain why we observed that the risk of stroke was higher in TBM patients with meningeal enhancement and VAFs. In addition, previous studies have reported that hydrocephalus was related to stroke in patients with TBM (12). However, most of the stroke lesions and hydrocephalus were observed on imaging during admission in these studies; therefore, the demonstration of a temporal relation cannot be achieved (12). In the present study, we excluded cases with a history of previous stroke, and the result demonstrated that hydrocephalus was a strong predictor of AIS within the 30-day follow-up. A further subgroup analysis suggested that the association remains significant, regardless of whether VAFs existed in TBM patients. Hydrocephalus may reduce blood flow by increasing intracranial pressure and dilating ventricles to stretch the blood vessel, which aggravates cerebral ischemia (8). This physical compression of the microvasculature probably interprets the association between hydrocephalus and AIS, which was independent of the VAFs (21).

In recent years, several predictive models have been developed for the prevention of vascular events in the general population, such as the Framingham Stroke Risk Profile and atherosclerotic cardiovascular disease risk stratification (17, 18). However, there are currently no risk assessment tools that perform a comprehensive assessment of vascular risk in TBM patients. A recent study confirmed that the Framingham risk score was higher in AIS patients with TBM, but the predictive performance remains unclear (8). In the present study, we added inflammation-associated neuroimaging markers to the stratification models to predict the risk of AIS. The predictive ability for the AIS outcome was improved and validated by increased C-statistic, NRI, and IDI. Future studies to develop predictive models should take into account the importance of these factors.

Currently, the use of prophylactic antiplatelet drugs for stroke prevention in the TBM population remains controversial. Our analysis revealed that the indirect effect of inflammation on functional disability through AIS is < 20%, which means that over 80% of functional damage results from other pathways to functional disability without AIS. Therefore, clinical therapies targeting inflammation and hydrocephalus should be given more attention in further studies to improve the functional outcome in TBM patients.

The strengths of this study include the large sample size of patients recruited from multi-centers with a robust analysis approach. We explored the underlying mechanism in stroke cases with and without VAFs and identified individuals with high risk for AIS in TBM. However, we realized that this study has the following limitations. First, the early definite diagnosis of TBM is difficult, and our study included patients with bacteriologically or clinically diagnosed TBM, which may lead to information bias. Second, it was an observational cohort study, and even though we used various analyses, including PSM and IPTW, to control the confounders, the unmeasured confounding factors might have biased our results. Third, due to the lack of vascular imaging evaluation of TBM patients in the clinical setting, it is impossible to further explore stroke subtypes for a better understanding of the pathogenesis. Fourth, our study was focused on the complications caused by stroke in TBM patients. Previous studies have shown that other TBM complications, such as myelitis (22), vision impairment (23), cognitive dysfunction (24), and seizure (25), similarly affect patients' quality of life. Long-term follow-up and neurological monitoring may be necessary for TBM patients. Finally, we found that AIS mediates < 20% of the association between neuroimaging markers of inflammation and functional disability. Future studies should pay more attention to novel anti-inflammatory treatments to improve the functional outcome (26).

## Conclusions

Our study provides evidence of the predictive factors of AIS in TBM patients. These findings implied the importance of assessing inflammation markers, which may contribute to improving AIS risk stratification in the TBM population. Future studies should pay more attention to clinical therapies targeting inflammation and hydrocephalus and functional outcomes.

## Data availability statement

The raw data supporting the conclusions of this article will be made available by the authors, without undue reservation.

## Ethics statement

The studies involving humans were approved by the Ethics Committee at Beijing Chest Hospital (Approval number: YJS-2022-07). The studies were conducted in accordance with the local legislation and institutional requirements. The ethics committee/institutional review board waived the requirement of written informed consent for participation from the participants or the participants' legal guardians/next of kin because Informed consent was exempted by the Ethics Committee because of the retrospective nature of this research.

## Author contributions

Y-JG: Data curation, Formal analysis, Investigation, Methodology, Software, Writing – original draft, Writing – review & editing. X-LG: Data curation, Formal analysis, Funding acquisition, Investigation, Writing – original draft, Writing – review & editing. R-YZ: Writing – original draft, Writing – review & editing. YL: Writing – original draft, Writing – review & editing. E-LW: Writing – original draft, Writing – review & editing. S-HL: Writing – original draft, Writing – review & editing. HJ: Supervision, Writing – original draft, Writing – review & editing. H-FD: Conceptualization, Supervision, Writing – original draft, Writing – review & editing. Z-ZY: Conceptualization, Supervision, Writing – original draft, Writing – review & editing. W-ML: Conceptualization, Funding acquisition, Methodology, Supervision, Writing – original draft, Writing – review & editing.
