# Supplementary material for: Acute ischemic stroke in tuberculous meningitis
Source: Front Public Health. 2024 Mar 21;12:1362465. doi: 10.3389/fpubh.2024.1362465 (PMC10991691; doi:10.3389/fpubh.2024.1362465)
Supplement: Supplementary file 1 [file Data_Sheet_1.docx]

**Figure S1 Study Flow chart**

**
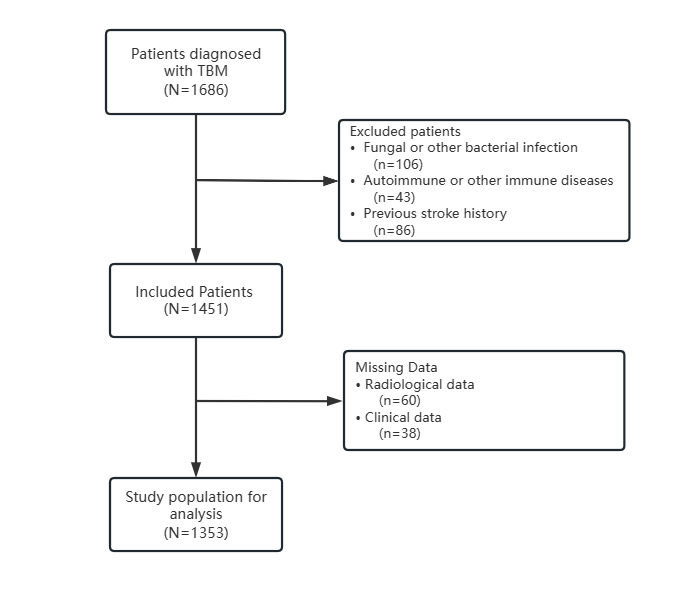
**

**Table S1 Hydrocephalus and non-hydrocephalus groups were well balanced after IPTW and PSM**

|  | Overall (n=1353) | Hydrocephalus | | | Standardised difference | Standardised difference (PSM) | Standardised difference (IPTW) |
| --- | --- | --- | --- | --- | --- | --- | --- |
| **Variables** |  | NO (n=1201) | YES(n=152) | P value |  |  |  |
| **Demographics** |  |  |  |  |  |  |  |
| Age, y | 38.8±18.3 | 38.9±18.4 | 38.2±18.0 | 0.541 | 0.040 | 0.008 | 0.076 |
| Men | 718 (53.0) | 652 (54.3) | 66 (43.4) | 0.011 | 0.287 | 0.176 | 0.182 |
| Body mass index, kg/m^2^ | 21.6±3.0 | 21.7±3.1 | 20.9±2.4 | 0.006 | 0.219 | 0.129 | 0.070 |
| **Vascular risk factors** |  |  |  |  |  |  |  |
| Hypertension | 185 (13.6) | 169 (14.1) | 16 (10.5) | 0.231 | 0.108 | 0.209 | 0.195 |
| Diabetes | 158 (11.6) | 140 (11.7) | 18 (11.8) | 0.947 | 0.006 | 0.198 | 0.174 |
| Hyperlipidemia | 151 (11.1) | 128 (10.7) | 23 (15.1) | 0.099 | 0.134 | 0.198 | 0.149 |
| Atrial fibrillation | 45 (3.3) | 44 (3.7) | 1(0.7) | 0.052 | 0.208 | 0.127 | 0.083 |
| Smoke | 85 (6.2) | 81 (6.7) | 4 (2.6) | 0.050 | 0.195 | 0.043 | 0.091 |
| **Clinical features** |  |  |  |  |  |  |  |
| Diagnosis classification |  |  |  | 0.003 | 0.341 | 0.173 | 0.044 |
| Probable TBM | 334 (24.6) | 296 (24.6) | 38 (25.0) |  |  |  |  |
| Possible TBM | 840 (62.0) | 733 (61.0) | 107 (70.4) |  |  |  |  |
| Definite TBM | 179 (13.2) | 172 (14.3) | 7 (4.6) |  |  |  |  |
| BMRC |  |  |  | <0.001 | 0.747 | 0.098 | 0.052 |
| Stage I | 683 (50.4) | 650 (54.1) | 33 (21.7) |  |  |  |  |
| Stage II | 495 (36.5) | 420 (35.0) | 75 (49.3) |  |  |  |  |
| Stage III | 175 (12.9) | 131 (10.9) | 44 (28.9) |  |  |  |  |
| **Cerebrospinal Fluid** |  |  |  |  |  |  |  |
| Pressure, mm H20 | 239.9±77.2 | 237.43±75.3 | 259.7±88.3 | 0.001 | 0.271 | 0.126 | 0.034 |
| Leukocyte count,10^6^/L | 203.8±257.3 | 206.2±251.8 | 184.21±296.9 | 0.320 | 0.080 | 0.115 | 0.195 |
| Glucose, mmol/L | 2.2±1.2 | 2.1±1.1 | 2.2±1.2 | 0.846 | 0.017 | 0.188 | 0.078 |
| Chloride, mmol/L | 112.9±7.6 | 113.0±7.4 | 111.8±9.2 | 0.058 | 0.149 | 0.065 | 0.012 |
| Protein, mg/dl | 155.6±92.4 | 148.5±87.0 | 211.4±113.0 | <0.001 | 0.623 | 0.032 | 0.067 |
| **Peripheral blood Findings** |  |  |  |  |  |  |  |
| White blood cell,10^9^/L | 7.4±2.3 | 7.3±2.9 | 8.3±3.2 | <0.001 | 0.338 | 0.183 | 0.092 |
| Neutrophil,10^9^/L | 5.5±2.1 | 5.4±2.8 | 6.6±3.1 | <0.001 | 0.427 | 0.130 | 0.013 |
| Monocyte,10^9^/L | 0.5±0.2 | 0.5±0.3 | 0.5±0.4 | 0.676 | 0.037 | 0.009 | 0.135 |
| Lymphocytes,10^9^/L | 1.2±0.5 | 1.2±0.8 | 1.1±0.5 | 0.010 | 0.248 | 0.165 | 0.021 |
| Platelet ,10^9^/L | 243.±65.0 | 243.9±84.3 | 237.6±92.0 | 0.039 | 0.071 | 0.039 | 0.050 |
| C-reactive protein, mg/L | 18.4±19.3 | 17.7±28.5 | 23.8±31.6 | 0.015 | 0.202 | 0.364 | 0.019 |
| **Treatment** |  |  |  |  |  |  |  |
| Isoniazid | 1306(96.5) | 1161(96.7) | 145(95.4) | 0.419 | 0.030 | 0.005 | 0.046 |
| Rifampicin | 1160(85.7) | 1028(85.6) | 132(86.8) | 0.679 | 0.060 | 0.028 | 0.092 |
| Pyrazinamide | 1321(97.6) | 1173(97.7) | 148(97.4) | 0.819 | 0.019 | 0.179 | 0.083 |
| Ethambutol | 1092(80.7) | 962(80.1) | 130(85.5) | 0.110 | 0.139 | 0.208 | 0.163 |
| Fluoroquinolones | 445(32.8) | 402(33.5) | 43(28.3) | 0.200 | 0.152 | 0.298 | 0.155 |
| Other anti-tuberculosis drugs | 189(13.9) | 165(13.7) | 24(15.8) | 0.492 | 0.033 | 0.006 | 0.059 |
| Intravenous dexamethasone | 792(58.5) | 702(58.5) | 90(59.2) | 0.858 | 0.012 | 0.218 | 0.198 |
| Intrathecal injection | 868(64.1) | 752(62.6) | 116(76.3) | 0.001 | 0.623 | 0.048 | 0.072 |
| Dehydration treatment | 1264(93.4) | 1112(92.6) | 152(100) | 0.001 | 0.526 | 0.102 | 0.079 |

BMRC, British Medical Research Council; PSM, propensity score matching; IPTW, inverse probability of treatment weighting.

**Table S2 Tuberculoma and non-tuberculoma groups were well balanced after IPTW and PSM**

|  | Overall (n=1353) | Tuberculoma | | | Standardised difference | Standardised difference (PSM) | Standardised difference (IPTW) |
| --- | --- | --- | --- | --- | --- | --- | --- |
| **Variables** |  | NO (n=765) | YES(n=588) | P value |  |  |  |
| **Demographics** |  |  |  |  |  |  |  |
| Age, y | 38.8±18.3 | 42.6±19.2 | 34.1±15.29 | <0.001 | 0.481 | 0.003 | 0.085 |
| Men | 718 (53.0) | 414±54.1 | 304±51.7 | 0.408 | 0.048 | 0.001 | 0.011 |
| Body mass index, kg/m^2^ | 21.6±3.0 | 21.51±3.03 | 21.75±3.04 | 0.157 | 0.078 | 0.060 | 0.048 |
| **Vascular risk factors** |  |  |  |  |  |  |  |
| Hypertension | 185 (13.6) | 124 (16.2) | 61 (10.4) | 0.003 | 0.173 | 0.007 | 0.049 |
| Diabetes | 158 (11.6) | 123 (16.1) | 35 (6.0) | <0.001 | 0.328 | 0.118 | 0.012 |
| Hyperlipidemia | 151 (11.1) | 81 (10.6) | 70 (11.9) | 0.500 | 0.042 | 0.046 | 0.029 |
| Atrial fibrillation | 45 (3.3) | 41 (5.4) | 4 (0.7) | <0.001 | 0.276 | <0.001 | 0.094 |
| Smoke | 85 (6.2) | 45 (5.9) | 40 (6.8) | 0.563 | 0.038 | 0.074 | 0.020 |
| **Clinical features** |  |  |  |  |  |  |  |
| Diagnosis classification |  |  |  | <0.001 | 0.681 | 0.135 | 0.228 |
| Probable TBM | 334 (24.6) | 189 (24.7) | 145 (24.7) |  |  |  |  |
| Possible TBM | 840 (62.0) | 407 (53.2) | 433 (73.6) |  |  |  |  |
| Definite TBM | 179 (13.2) | 169 (22.1) | 10 ( 1.7) |  |  |  |  |
| BMRC |  |  |  | <0.001 | 0.278 | 0.015 | 0.056 |
| Stage I | 683 (50.4) | 398 (52.0) | 285 (48.5) |  |  |  |  |
| Stage II | 495 (36.5) | 244 (31.9) | 251 (42.7) |  |  |  |  |
| Stage III | 175 (12.9) | 123 (16.1) | 52 (8.8) |  |  |  |  |
| **Cerebrospinal Fluid** |  |  |  |  |  |  |  |
| Pressure, mm H20 | 239.9±77.2 | 236.2±77.7 | 244.8±76.3 | 0.043 | 0.111 | 0.055 | 0.014 |
| Leukocyte count,10^6^/L | 203.8±257.3 | 219.3±271.2 | 183.5±236.6 | 0.011 | 0.141 | 0.004 | 0.004 |
| Glucose, mmol/L | 2.2±1.2 | 2.22±1.2 | 2.1±1.1 | 0.144 | 0.081 | 0.039 | 0.038 |
| Chloride, mmol/L | 112.9±7.6 | 112.5±7.6 | 113.3±7.6 | 0.062 | 0.102 | 0.030 | 0.017 |
| Protein, mg/dl | 155.6±92.4 | 168.5±96.7 | 138.8±83.7 | <0.001 | 0.328 | 0.114 | 0.033 |
| **Peripheral blood Findings** |  |  |  |  |  |  |  |
| White blood cell,10^9^/L | 7.4±2.3 | 7.2±3.0 | 7.6±3.0 | 0.016 | 0.132 | 0.081 | 0.016 |
| Neutrophil,10^9^/L | 5.5±2.1 | 5.41±2.8 | 5.7±2.7 | 0.055 | 0.106 | 0.062 | 0.016 |
| Monocyte,10^9^/L | 0.5±0.2 | 0.50±0.3 | 0.5±0.5 | 0.004 | 0.154 | 0.073 | 0.014 |
| Lymphocytes,10^9^/L | 1.2±0.5 | 1.22±0.6 | 1.2±0.8 | 0.762 | 0.016 | 0.044 | 0.020 |
| Platelet ,10^9^/L | 243.2±65.0 | 235.5±81.2) | 253.1 (89.1) | <0.001 | 0.206 | 0.027 | 0.027 |
| C-reactive protein, mg/L | 18.4±19.3 | 19.0 (30.9) | 17.6 (26.1) | 0.367 | 0.050 | 0.020 | 0.012 |
| **Treatment** |  |  |  |  |  |  |  |
| Isoniazid | 1306(96.5) | 729(95.3) | 577(98.1) | 0.005 | 0.138 | 0.065 | 0.029 |
| Rifampicin | 1160(85.7) | 654(85.5) | 506(86.1) | 0.769 | 0.017 | 0.045 | 0.035 |
| Pyrazinamide | 1321(97.6) | 737(96.3) | 584(99.3) | <0.001 | 0.206 | 0.041 | 0.052 |
| Ethambutol | 1092(80.7) | 615(80.4) | 477(81.1) | 0.736 | 0.031 | 0.059 | 0.038 |
| Fluoroquinolones | 445(32.8) | 253(33.1) | 192(32.7) | 0.871 | 0.022 | 0.216 | 0.199 |
| Other anti-tuberculosis drugs | 189(13.9) | 106(13.9) | 83(14.1) | 0.891 | 0.018 | 0.169 | 0.081 |
| Intravenous dexamethasone | 792(58.5) | 451(59.0) | 341(58.0) | 0.722 | 0.060 | 0.038 | 0.096 |
| Intrathecal injection | 868(64.1) | 472(61.7) | 396(67.3) | 0.032 | 0.112 | 0.066 | 0.075 |
| Dehydration treatment | 1264(93.4) | 713(93.2) | 551(93.7) | 0.710 | 0.019 | 0.043 | 0.048 |

BMRC, British Medical Research Council; PSM, propensity score matching; IPTW, inverse probability of treatment weighting.

**Table S3 Meningeal enhancement and non-meningeal enhancement groups were well balanced after IPTW and PSM**

|  | Overall (n=1353) | Meningeal enhancement | | | Standardised difference | Standardised difference (PSM) | Standardised difference (IPTW) |
| --- | --- | --- | --- | --- | --- | --- | --- |
| **Variables** |  | NO (n=990) | YES(n=363) | P value |  |  |  |
| **Demographics** |  |  |  |  |  |  |  |
| Age, y | 38.8±18.3 | 40.2±18.9 | 35.0±15.9 | <0.001 | 0.299 | 0.010 | 0.053 |
| Men | 718 (53.0) | 514 (51.9) | 204 (56.2) | 0.182 | 0.086 | 0.032 | 0.019 |
| Body mass index, kg/m^2^ | 21.6±3.0 | 21.5±3.0 | 21.6±3.0 | 0.577 | 0.034 | 0.074 | 0.015 |
| **Vascular risk factors** |  |  |  |  |  |  |  |
| Hypertension | 185 (13.6) | 145 (14.6) | 40 (11.0) | 0.103 | 0.109 | 0.019 | 0.017 |
| Diabetes | 158 (11.6) | 138 (13.9) | 20 (5.5) | <0.001 | 0.287 | 0.061 | 0.065 |
| Hyperlipidemia | 151 (11.1) | 103 (10.4) | 48 (13.2) | 0.173 | 0.087 | 0.009 | 0.091 |
| Atrial fibrillation | 45 (3.3) | 37 (3.7) | 8 (2.2) | 0.221 | 0.090 | 0.020 | 0.093 |
| Smoke | 85 (6.2) | 66 (6.7) | 19 (5.2) | 0.403 | 0.061 | 0.093 | 0.054 |
| **Clinical features** |  |  |  |  |  |  |  |
| Diagnosis classification |  |  |  | <0.001 | 0.458 | 0.077 | 0.015 |
| Probable TBM | 334 (24.6) | 223 (22.5) | 111 (30.6) |  |  |  |  |
| Possible TBM | 840 (62.0) | 601 (60.7) | 239 (65.8) |  |  |  |  |
| Definite TBM | 179 (13.2) | 166 (16.8) | 13 (3.6) |  |  |  |  |
| BMRC |  |  |  | 0.001 | 0.222 | 0.094 | 0.084 |
| Stage I | 683 (50.4) | 525 (53.0) | 158 (43.5) |  |  |  |  |
| Stage II | 495 (36.5) | 334 (33.7) | 161 (44.4) |  |  |  |  |
| Stage III | 175 (12.9) | 131 (13.2) | 44 (12.1) |  |  |  |  |
| **Cerebrospinal Fluid** |  |  |  |  |  |  |  |
| Pressure, mm H20 | 239.9±77.2 | 234.6±77.3 | 254.4±75.1 | <0.001 | 0.260 | 0.074 | 0.053 |
| Leukocyte count,10^6^/L | 203.8±257.3 | 200.8±256.3 | 211.7±260.2 | 0.489 | 0.042 | 0.054 | 0.069 |
| Glucose, mmol/L | 2.2±1.2 | 2.2±1.2 | 2.0±0.9 | 0.001 | 0.220 | 0.062 | 0.006 |
| Chloride, mmol/L | 112.9±7.6 | 112.8±7.5 | 113.0±8.0 | 0.753 | 0.019 | 0.121 | 0.022 |
| Protein, mg/dl | 155.6±92.4 | 157.9±97.2 | 149.2±77.6 | 0.125 | 0.099 | 0.156 | 0.122 |
| **Peripheral blood Findings** |  |  |  |  |  |  |  |
| White blood cell,10^9^/L | 7.4±2.3 | 7.2±2.9 | 7.7±3.2 | 0.002 | 0.183 | 0.058 | 0.049 |
| Neutrophil,10^9^/L | 5.5±2.1 | 5.4±2.7 | 5.7±2.9 | 0.097 | 0.101 | 0.012 | 0.048 |
| Monocyte,10^9^/L | 0.5±0.2 | 0.5±0.3 | 0.5±0.6 | 0.001 | 0.174 | 0.160 | 0.025 |
| Lymphocytes,10^9^/L | 1.2±0.5 | 1.1±0.6 | 1.3±0.9 | <0.001 | 0.207 | 0.074 | 0.002 |
| Platelet ,10^9^/L | 243.2±65.0 | 234.1±84.1 | 267.8±83.3 | <0.001 | 0.402 | 0.008 | 0.025 |
| C-reactive protein, mg/L | 18.4±19.3 | 18.8±29.5 | 17.4±27.3 | 0.431 | 0.049 | 0.056 | 0.017 |
| **Treatment** |  |  |  |  |  |  |  |
| Isoniazid | 1306(96.5) | 954(96.4) | 352(97.0) | 0.590 | 0.033 | 0.028 | 0.019 |
| Rifampicin | 1160(85.7) | 830(83.8) | 330(90.9) | 0.001 | 0.226 | 0.061 | 0.073 |
| Pyrazinamide | 1321(97.6) | 958(96.8) | 363(100) | 0.001 | 0.282 | 0.042 | 0.057 |
| Ethambutol | 1092(80.7) | 786(79.4) | 306(84.3) | 0.043 | 0.106 | 0.037 | 0.048 |
| Fluoroquinolones | 445(32.8) | 297(30.0) | 148(40.8) | <0.001 | 0.183 | 0.064 | 0.069 |
| Other anti-tuberculosis drugs | 189(13.9) | 137(13.8) | 52(14.3) | 0.819 | 0.015 | 0.116 | 0.094 |
| Intravenous dexamethasone | 792(58.5) | 585(59.1) | 207(57.0) | 0.494 | 0.052 | 0.073 | 0.071 |
| Intrathecal injection | 868(64.1) | 626(63.2) | 242(66.7) | 0.243 | 0.092 | 0.086 | 0.093 |
| Dehydration treatment | 1264(93.4) | 917(92.6) | 347(95.6) | 0.051 | 0.126 | 0.045 | 0.051 |

BMRC, British Medical Research Council;PSM, propensity score matching; IPTW, inverse probability of treatment weighting.

**Table S4. The Prevalence of the cerebrovascular risk identified by the stroke risk stratification models**

|  | Cerebrovascular Risk, % | | |
| --- | --- | --- | --- |
|  | Low risk | Medium risk | High risk |
| Model 1 | 71.47 (68.97-73.85) | 14.86 (13.03-16.89) | 13.67 (11.91-15.64) |
| Model 2 | 56.25 (53.56-58.91) | 32.15 (29.68-34.72) | 11.60 (9.97-13.46) |
| Model 3 | 65.71 (63.10-68.23) | 24.17 (21.93-26.56) | 10.13 (8.60-11.89) |

Model 1, Atherosclerotic Cardiovascular Disease Risk Model recommended by European Society Of Cardiology;

Model 2, Stroke risk score card recommended by Stroke Prevention Committee of the Chinese National Health Commission;

Model 3, Framingham Stroke Risk Profile.
